# miR-889-3p Facilitates the Browning Process of White Adipocyte Precursors by Targeting the *SON* Gene

**DOI:** 10.3390/ijms242417580

**Published:** 2023-12-17

**Authors:** Wenqiang Sun, Xiaoxiao Zhang, Xue Bai, Kun Du, Li Chen, Haoding Wang, Xianbo Jia, Songjia Lai

**Affiliations:** 1 State Key Laboratory of Swine and Poultry Breeding Industry, College of Animal Science and Technology, Sichuan Agricultural University, Chengdu 611134, China; wqsun2021@163.com (W.S.); zxx18137928846@163.com (X.Z.); baixue333work@163.com (X.B.); dukun1672@163.com (K.D.); chenl2020302120@163.com (L.C.); 19848671707@163.com (H.W.); jaxb369@sicau.edu.cn (X.J.); 2 Key Laboratory of Livestock and Poultry Multi-Omics, Ministry of Agriculture and Rural Affairs, College of Animal Science and Technology, Sichuan Agricultural University, Chengdu 611134, China; 3 Farm Animal Genetic Resources Exploration and Innovation Key Laboratory of Sichuan Province, Sichuan Agricultural University, Chengdu 611134, China

**Keywords:** miR-889-3p, white adipocyte, browned tissue, *SON*, UCP1

## Abstract

It is well-established that beige/brown adipose tissue can dissipate stored energy through thermogenesis; hence, the browning of white adipocytes (WAT) has garnered significant interest in contemporary research. Our preceding investigations have identified a marked downregulation of miR-889-3p concurrent with the natural maturation of brown adipose tissue. However, the specific role and underlying molecular mechanisms of miR-889-3p in the browning process of white adipose tissue warrant further elucidation. In this research, we initially delved into the potential role of miR-889-3p in preadipocyte growth via flow cytometry and CCK-8 assay, revealing that miR-889-3p can stimulate preadipocyte growth. To validate the potential contribution of miR-889-3p in the browning process of white adipose tissue, we established an in vitro rabbit white adipocyte browning induction, which exhibited a significant upregulation of miR-889-3p during the browning process. RT-qPCR and Western blot analysis indicated that miR-889-3p overexpression significantly amplified the mRNA levels of UCP1, PRDM16, and CIDEA, as well as UCP1 protein levels. Furthermore, miR-889-3p overexpression fostered intracellular triglyceride accumulation. Conversely, the downregulation of miR-889-3p hindered the browning of rabbit preadipocytes. Subsequently, based on target gene prediction and luciferase reporter gene determination, we demonstrated that miR-889-3p directly targets the 3′-UTR region of *SON*. Lastly, we observed that inhibiting *SON* could facilitate the browning of rabbit preadipocytes. In conclusion, our findings suggest that miR-889-3p facilitates the browning process of white adipocyte precursors by specifically targeting the *SON* gene.

## 1. Introduction

Traditionally, it was believed that the body contained two types of adipose tissue. The first type, white adipose tissue (WAT), originates from Myogenic regulatory factor 5 negative (Myf5-) progenitor cells and stores energy as triglycerides [1]. The second type, brown adipose tissue, differentiates from Myogenic regulatory factor 5 positive (Myf5+) progenitor cells and highly expresses uncoupling protein 1 (UCP1), which is intimately linked to thermogenesis [2]. Beyond these conventional white and brown adipose tissues, research in 1992 discovered the existence of beige adipocytes within the white fat pool [3]. These beige adipocytes, like brown adipocytes, differentiate from Myf5- progenitor cells [3]. They contain numerous small oil droplets, have significantly more mitochondria compared to white adipocytes, and express the UCP1 gene [3]. Therefore, beige adipocytes are also called brown adipocytes. Non-shivering thermogenesis in mammals is conducted not only by classical brown adipocytes but also by these beige adipocytes [4,5]. Furthermore, the stimulation of beige adipocyte activity is linked to weight loss, insulin resistance reduction, and obesity prevention [6,7]. Hence, amplifying the number or activity of thermogenic adipocytes could potentially manipulate fat plasticity and enhance metabolic health. Understanding the molecular mechanism behind WAT browning regulation is therefore of paramount importance.

Small non-coding RNA (miRNA) can regulate gene expression at the post-transcriptional level. Recent studies have highlighted miRNA as a crucial factor in regulating white adipocyte browning [8]. However, research on miR-889-3p has primarily focused on tumor and cancer-related fields. For instance, Zhu et al. found that miR-889-3p inhibits lung cancer cell proliferation and epithelial–mesenchymal transformation by downregulating homologous domain interacting protein kinase 1 (HIPK1) [9]. Zhao et al. discovered that the overexpression of miR-889-3p can target and inhibit HOXB7, thereby impeding cervical cancer cell proliferation, migration, and invasion; enhancing cell apoptosis and radiosensitivity; and weakening tumor growth in vitro and in vivo [10]. However, studies on miR-889-3p in fat thermogenesis are limited, indicating the need for future research to elucidate its potential positive role in this process.

The *SON* DNA-binding protein (*SON*) is a widely expressed 2426-amino acid protein found in nuclear speckles featuring an RNA-binding G-patch domain, a double-stranded RNA binding motif, a forkhead N-terminal region, an arginine/serine-rich region, and amino acid repeats [11]. It facilitates the efficient splicing of challenging transcripts, playing a key role in cell cycle, DNA repair, apoptosis, and epigenetic modification [11]. Essential for cellular functions and genomic stability, *SON* also maintains the nuclear organization and function of pre-mRNA processing factors; its disruption can lead to cell cycle issues [12]. Previous studies on the *SON* protein have focused mainly on cancer [13], with not much attention given to its role in fat metabolism. Therefore, it would be valuable to explore what role *SON* might play in this area.

In this research, we initially examined the expression of miR-889-3p in brown adipose tissues. Using synthetic mimics and inhibitors of miR-889-3p, we characterized its role in browning induction by observing changes in the mRNA and protein expression of heat-producing marker genes and Oil Red O staining. The potential interaction mechanism between miR-889-3p and *SON* was explored through bioinformatics analysis and luciferase reporter gene determination. Finally, we knocked down *SON* using siRNA to further verify its role in the browning of white adipocytes.

## 2. Results

### 2.1. miR-889-3p Promotes Rabbit Preadipocyte Proliferation

**Figure 1:**
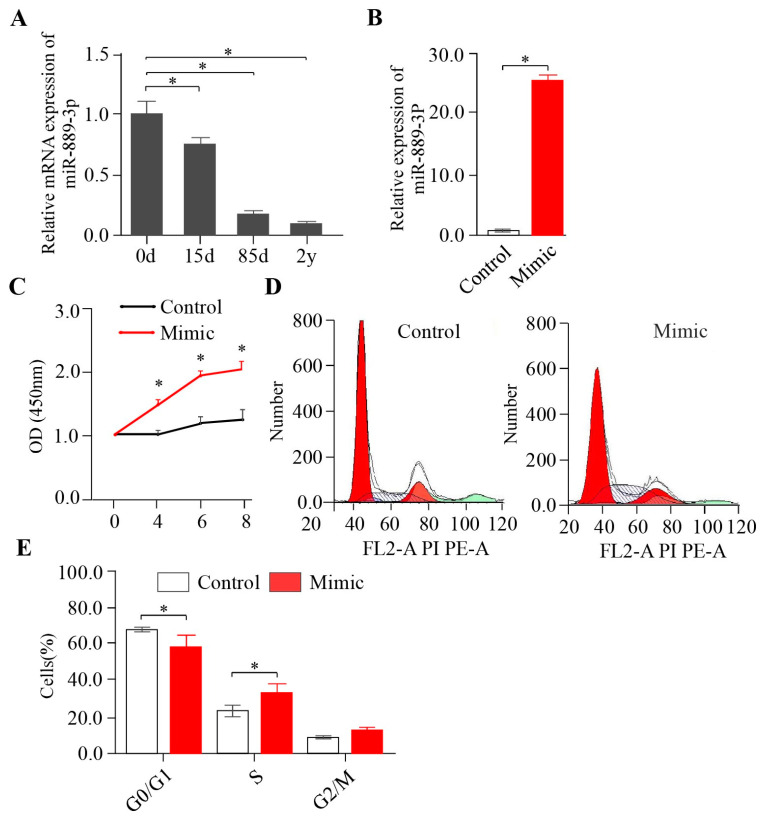
Effect of miR-889-3p on proliferation of rabbit white precursor adipocytes. (**A**) Relative expression of miR-889-3p in white and brown tissues. (**B**) Cell viability after overexpression of miR-889-3p. (*n* = 5). (**C**) Transfection efficiency of miR-889-3p. (**D**,**E**) Cell cycle changes by flow cytometry and digital conversion histogram. The red color represents the miR-889-3p mimic, while white represents the control group. The difference was significant (*p < 0.05).

In a prior study, we collected brown fat from the interscapular of Tianfu black rabbits at 0 days, 15 days, 85 days, and 2 years. We observed that the brown fat tissue at 0 days exhibited a classical brown fat structure with multi-chambered and small fat droplets, while the interscapular fat tissue at 2 years demonstrated a typical white fat structure with a single chamber and large fat droplets [14]. Intriguingly, according to our previously performed RNA-seq data, miR-889-3p was significantly downregulated during the natural growth of brown fat [14]. This finding was corroborated by PCR results (Figure 1A). These observations suggest that miR-889-3p may play a role in the browning of white adipose tissue. However, existing research on miR-889-3p primarily centers on tumor- and cancer-related fields. The effect of miR-889-3p on adipose tissue is still unknown. Our initial step was to investigate the evolutionary conservation of miR-889-3p across different species. Interestingly, we found that the sequence of miR-889-3p was 100% conserved (Figure S1). To delve into the role of miR-889-3p within adipose tissue, we first transfected miR-889-3p into white precursor adipocytes and assessed its transfection efficiency. RT-qPCR results revealed that the expression of miR-889-3p in the mimic group was significantly elevated compared to the control group (Figure 1B). CCK-8 assay results demonstrated that cell viability significantly improved following the overexpression of miR-889-3p (Figure 1C). Furthermore, cell cycle analysis via flow cytometry indicated that the overexpression of miR-889-3p resulted in fewer cells being stalled in the G0/G1 phase and more cells progressing to the S phase (Figure 1D,E). These findings suggest that miR-889-3p can stimulate rabbit preadipocyte proliferation.

### 2.2. Establishment of the Browning Induction of Rabbit White Preadipocytes In Vitro

**Figure 2:**
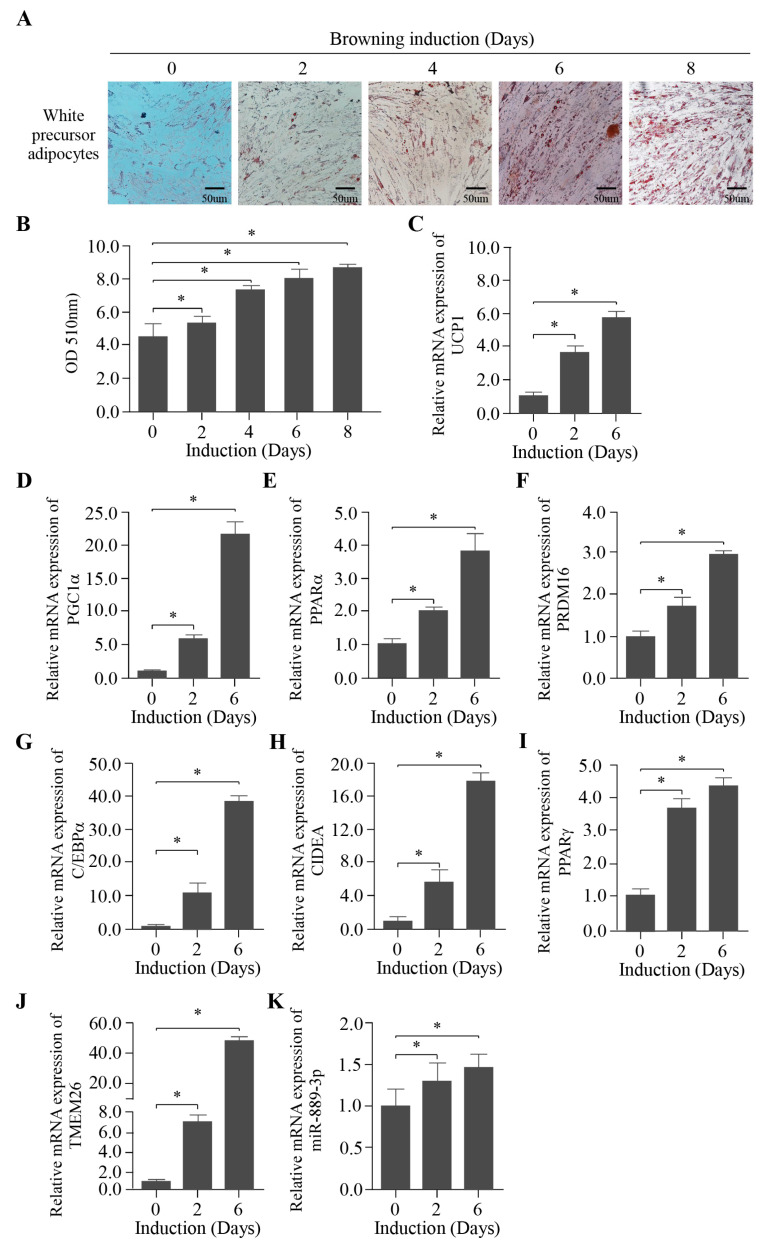
Establishment of browning induction of rabbit white preadipocytes invitro. (**A**) Oil Red O staining pictures of cells on days 0, 2, 4, 6, and 8 of browning induction. (**B**) Quantitative results of Oil Red O staining. (**C**–**J**) Expression of thermogenic marker genes (UCP1, PPARα, PGC1α, PRDM16, C/EBPα, CIDEA, PPARα, and TMEM26) in browning induction. (**K**) Expression of miR-889-3p in browning induction. The difference was significant (*p < 0.05).

To investigate whether miR-889-3p plays a role in the browning of white adipose tissue, we initially established an in vitro browning induction of rabbit white preadipocytes. As depicted in Figure 2A, with the progression of browning induction days, the cell morphology gradually transitioned from spindle-shaped to slender. The lipid droplets within the cells increased over time, and the garland-like lipid droplets around the nucleus clustered together, distributing evenly across the cell culture plate. The quantitative results of Oil Red O staining are presented in Figure 2B. During browning induction, the absorbance (at OD value = 510 nm) displayed a significant upward trend. Moreover, the expression of thermogenic marker genes UCP1, PPARα, PGC1α, PRDM16, C/EBPα, CIDEA, PPARα, and TMEM26 were significantly upregulated, as confirmed by RT-qPCR (Figure 2C–J). These findings suggest that the browning induction of rabbit white preadipocytes was successfully established. Notably, miR-889-3p was also significantly upregulated during browning induction (Figure 2K).

### 2.3. The Overexpression of miR-889-3p Promotes the Browning of Rabbit Precursor White Adipocytes

**Figure 3:**
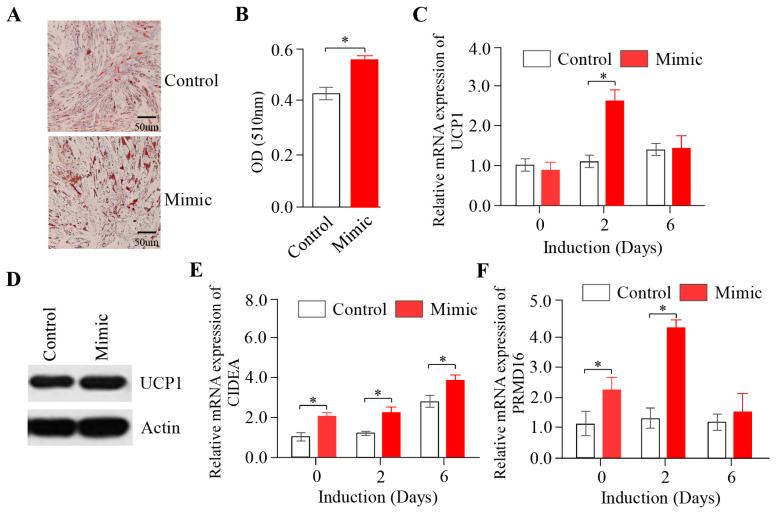
Effect of overexpression of miR-889-3p on browning of rabbit precursor white adipocytes. (**A**) Oil Red O staining results of miR-889-3p mimic and control. (**B**) Oil Red O quantification results. (**C**) Expression of UCP1 after transfection with mimic and control. (**D**) Protein content results of UCP1 in cells on the third day of induction after transfection with mimic and control by Western blotting. (**E**,**F**) Expression of CIDEA and PRDM16 after transfection with mimic and control. The red color represents the miR-889-3p mimic, while white represents the control group. The difference was significant (*p < 0.05).

To examine the impact of miR-889-3p on the browning of rabbit white precursor adipocytes, we transfected miR-889-3p into the cells. The Oil Red O staining results, displayed in Figure 3A, revealed that the number of fat droplets in the mimic group was significantly greater than that in the control group, with a notable increase in the number of large fat droplets. The quantification of fat droplets aligned with these observations (Figure 3B). As shown in Figure 3C,E,F, thermogenic marker genes such as UCP1, PPARα, and PRDM16 were significantly upregulated in the overexpression group compared to the control group during browning induction. Further protein analysis by Western blotting showed that UCP1 protein expression in the mimic group surpassed that in the control group (Figure 3D). These findings suggest that the overexpression of miR-889-3p can facilitate the browning of rabbit precursor white adipocytes.

### 2.4. The Inhibition of miR-889-3p Expression Inhibits the Browning of Rabbit Precursor White Adipocytes

**Figure 4:**
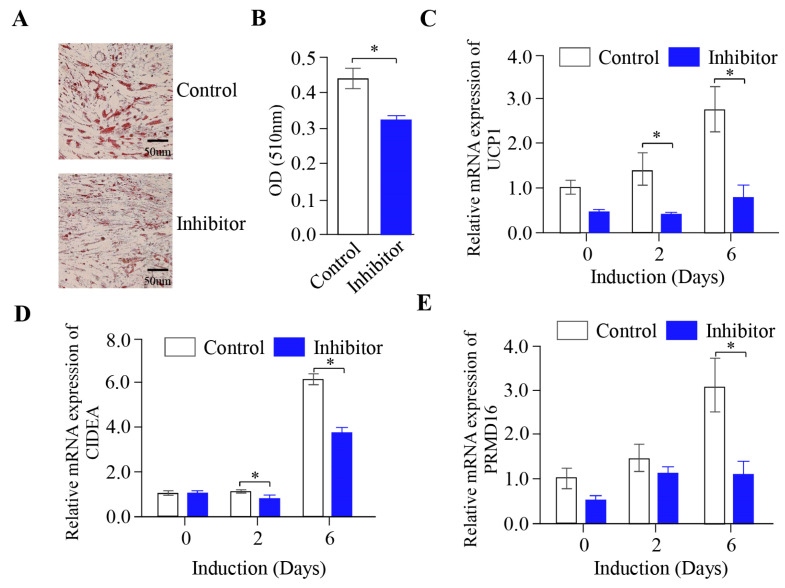
Effect of inhibition of miR-889-3p on browning of rabbit precursor white adipocytes. (**A**) Oil Red O staining results of miR-889-3p inhibitor and control. (**B**) Oil Red O quantification results. (**C**–**E**) Expression of UCP1, CIDEA, and PRDM16 after transfection with inhibitor and control. The blue color represents the miR-889-3p inhibitor, while white represents the control group. The difference was significant (*p < 0.05).

To further validate the influence of miR-889-3p on the browning of rabbit white precursor adipocytes in vitro, we performed transfection with a synthetic miR-889-3p inhibitor to suppress the expression of endogenous miR-889-3p. Following transfection, the number of fat droplets in the inhibitor group was significantly fewer than that in the inhibitor control group (Figure 4A), a finding consistent with the results of fat droplet quantification (Figure 4B). On the sixth day of browning induction, the expression of UCP1, PPARα, and PRDM16 mRNA in the inhibitor group was significantly lower than that in the inhibitor control group (Figure 4C–E). These findings suggest that inhibiting miR-889-3p expression impedes the browning of rabbit precursor white adipocytes.

### 2.5. The Effect of Inhibiting the SON Gene on the Browning of Rabbit White Precursor Adipocytes In Vitro

**Figure 5:**
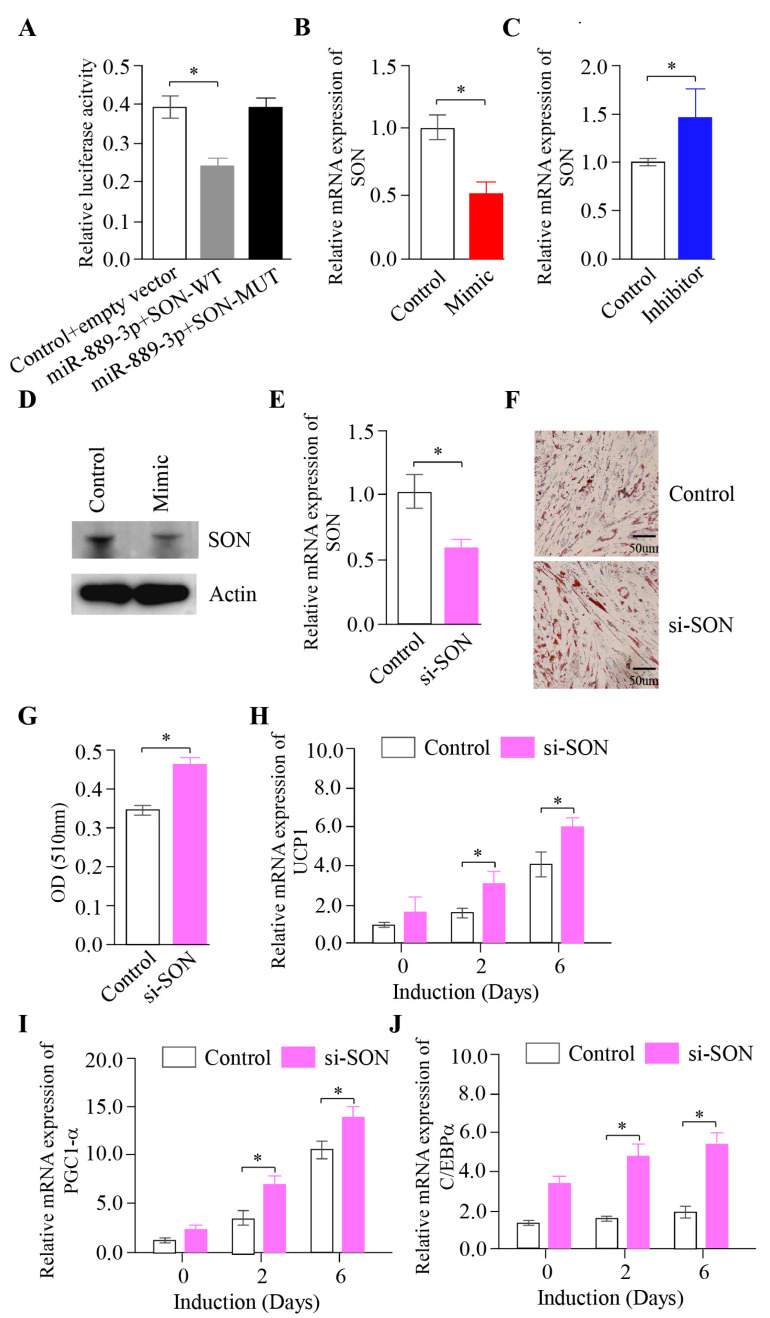
Effect of inhibiting *SON* gene on browning of rabbit white precursor adipocytes in vitro. (**A**) Luciferase assays were performed by co-transfection of *SON* WT and mutant plasmids with miR-8889-3p mimic and control (**B**,**C**) Expression of *SON* transfection with miR-889-3p mimic and inhibitor, respectively. (**D**) Protein content results of *SON* in cells after transfection with mimic and control by Western blotting. (**E**) Transfection efficiency of si-*SON*. (**F**) Oil Red O staining results of si-*SON* and NC. (**G**) Oil Red O quantification results. (**H**–**J**) Expression of UCP1, PGC1α, and C/EBPα after transfection with si-*SON* and control. The red color represents the miR-889-3p mimic, blue represents the miR-889-3p inhibitor, pink represents si-SON, and white represents the control group. The difference was significant (*p < 0.05).

To uncover the potential mechanism of miR-889-3p in the browning of rabbit precursor white adipocytes, we predicted the target gene of miR-889-3p using the miRanda. Our findings suggested that the *SON* gene could be a potential target. The luciferase reporter assay indicated that miR-889-3p could bind to the *SON* 3′UTR-WT region, but not to the *SON* 3′UTR-MUT region (Figure 5A). Furthermore, the overexpression of miR-889-3p could suppress *SON* gene expression, while the inhibition of miR-889-3p could enhance *SON* gene expression (Figure 5B,C). Further protein analysis by Western blotting showed that *SON* protein expression in the mimic group surpassed that in the control group (Figure 5D). These findings imply that *SON* is a target of miR-889-3p.

To examine the influence of *SON* on the browning of rabbit white precursor adipocytes, we utilized synthetic siRNA to inhibit endogenous *SON*. PCR results demonstrated that the *SON* gene content in the si-*SON* group significantly decreased when the *SON* interference analog was transfected, and browning was induced on the third day (Figure 5E). Oil Red O staining indicated that the quantity of lipid droplets in the si-*SON* group was significantly greater than that in the control group (Figure 5F). The quantification outcomes were in line with the staining results, with the si-*SON* group significantly surpassing the control group (Figure 5G). Thermogenic marker gene mRNA expression was assessed by RT-qPCR. The results revealed that UCP1, PGC1α, and CEBPA in the si-*SON* group were significantly higher than those in the control group on the second and sixth days of browning (Figure 5H–J). Taken together, these findings suggest that miR-889-3p can enhance white adipose browning by targeting *SON*.

## 3. Discussion

In this study, we gathered primary white fat cells from around the kidneys of rabbits and utilized DEX, Ins, IBMX, Rosi, T3, and ISO to induce the browning of white fat precursor cells. The induction method followed in this experiment was based on the browning induction method of Asano et al. [15] The browning of white precursor adipocytes essentially results from a highly sequential and precise regulation of gene expression, indicating that changes in the expression of specific genes can determine the adipocyte browning process. Hence, quantitatively detecting changes in the expression of thermogenic marker genes is a critical means of monitoring the browning process of white precursor adipocytes. In this experiment, we utilized RT-qPCR to measure the expression levels of thermogenic marker genes UCP1, PPARα, C/EBPα, PGC1α, and CIDEA at 0, 2, and 6 days, respectively. We found a significant upregulation in the expression of these thermogenic marker genes, consistent with previous research findings. The experimental results showed that the mRNA levels of heat-producing marker genes UCP1, CIDEA, PGC1α, and C/EBPα detected by PCR were significantly upregulated, aligning with the browning results of 3T3-L1 cells induced by Asano et al. [15]. Brown adipocytes are characterized by a high number of mitochondria within them, a trait shared by beige fat induced from white fat [16]. The mitochondrial membrane contains UCP1, which is almost exclusive to brown and beige fat cells. UCP1 is a proton transport protein that enables protons to leak through the mitochondrial inner membrane and dissipate and is typically used for the ATP synthesis of electrochemical gradients [17]. Over 50% of cell respiration in brown and beige adipocytes is related to the ATP synthesis electrochemical gradient [16,18]. It is noteworthy that compared to white fat, PGC1α is highly expressed in brown fat, which is primarily associated with cAMP-dependent thermogenesis in brown fat [19,20]. cAMP is the key second messenger of brown fat function and stimulates the level of key thermogenic factors. Hence, PGC1α can regulate mitochondrial biogenesis, adaptive thermogenesis, and oxidative metabolism in brown and beige fat. It associates with the PPARα complex and activates UCP1 expression by binding to PPARα response elements in the UCP1 promoter [21]. Moreover, PGC1α can activate the adaptive thermogenic gene program when expressed in white adipocytes [22,23].

Through transcriptome analysis, Seale et al. demonstrated that the expression level of C/EBPα in a brown fat pool was lower than that in a white fat pool. However, during the construction of this experimental induction, C/EBPα expression was significantly upregulated. This may suggest that browning induction did not impede the differentiation process of white adipocytes but occurred concurrently with browning [24]. Compared to white adipocytes, PRDM16 is selectively expressed in brown adipocytes and can enhance mitochondrial gene expression and mitochondrial density. The expression of UCP1 and PGC1α is further enhanced by cAMP, which strengthens the typical characteristic of brown adipocytes: uncoupled respiration. Importantly, PRDM16 activates the brown fat differentiation process in cultured white preadipocytes [24].CIDEA is a lipid droplet-related protein. Previous studies have shown that CIDEA is highly expressed in brown and beige adipocytes, but it was unexpectedly found that CIDEA acts as a heat-generating inhibitor at the molecular level [25]. Therefore, the results demonstrated that white preadipocytes treated with Dex, Ins, IBMX, Rosi, T3, and ISO were closer to mature beige adipocytes and had undergone a certain degree of browning.

miR-889-3p acts as a promoter of preadipocyte browning. Increasing research evidence indicates that miRNA, a type of short-chain non-coding RNA, can regulate the browning process of preadipocytes by targeting key genes that control white precursor fat [26,27,28,29,30]. In this study, we initially established an in vitro browning induction of rabbit white precursor adipocyte and measured the expression levels of miR-889-3p in the browning induction. The findings revealed a significant upregulation trend in miR-889-3p expression within the browning induction. Consequently, we further investigated the specific mechanism of miR-889-3p in the in vitro browning process of rabbit white precursor adipocytes. In previous research, Tan et al. discovered that following the overexpression of miR-669a-5p, there was a significant increase in UCP1, PGC1α, and other genes at both mRNA and protein levels. Therefore, they proposed that miR-669a-5p could promote preadipocyte browning [31]. Sun et al. suggested that miR-27 could inhibit preadipocyte browning by targeting the expression of PRDM16, PPARα, and PGC1α [32]. In our study, the overexpression of miR-889-3p demonstrated an increase in lipid droplets through Oil Red O staining. This was accompanied by an increase in the expression of thermogenic marker genes UCP1, CIDEA, and PRDM16 at the mRNA level. Western blot results showed a corresponding increase in the expression of UCP1 at the protein level. Conversely, the inhibition of miR-889-3p expression yielded opposite results. Thus, we posit that miR-889-3p acts as a promoter of preadipocyte browning.

In this study, the *SON* gene was confirmed to be the target gene of miR-889-3p through the dual-luciferase reporter gene method. The findings demonstrated that miR-889-3p could negatively regulate the expression of the *SON* gene. *SON* is an RNA-binding protein that acts as an mRNA splicing cofactor by promoting the efficient splicing of transcripts that possess weak splice sites [33]. Prior research on *SON* primarily focused on its role in cancer [34,35,36,37]. However, recent studies have revealed that when *SON* expression was knocked down, there was a significant decrease in viral particles colocalized in the late endosome labeled by CD63 (cluster differentiation 36) [38]. CD36 is a scavenger receptor involved in the high-affinity tissue uptake of long-chain fatty acids (FAs), and it promotes lipid accumulation and metabolic dysfunction under conditions of excessive fat supply [39]. Febbraio et al., in their study of CD36-deficient mice, found that these mice had normal lifespans, but fasting levels of cholesterol, non-esterified free fatty acids, and triacylglycerol were significantly elevated [40]. Thus, *SON* may play an indirect role in regulating the browning process of precursor adipocytes.

## 4. Materials and Methods

### 4.1. Animal Tissues and Sequencing

Male rabbit interscapular fat was taken for 0, 15, and 85 days and 2 years, respectively. The fat was sequenced by microRNA to detect the content of miR-889-3p in the tissue. The surplus tissue in liquid nitrogen was ground for the subsequent extraction of tissue cDNA and the content of miR-889-3p was detected by qPCR.

### 4.2. Cell Culture and Browning Induction

The primary preadipocytes were collected in the perirenal area of male Tianfu black rabbits using the method described earlier [41]. Briefly, preadipocytes were harvested from the perirenal adipose tissue of Tianfu black rabbits under sterile conditions. The tissues were immersed in PBS with 4% penicillin-streptomycin (Gibco) in a 6-well plate, with connective tissues and blood vessels meticulously excised. After triple washing with PBS, the adipose samples were enzymatically dissociated in a 15 mL centrifuge tube with 0.1% type I collagenase (Gibco, Carlsbad, CA, USA) at 37 °C for 1 h, with agitation at 15 min intervals. Digestion was neutralized with an equal volume of complete growth medium, followed by sequential filtration through 40 nm and 70 nm cell sieves. The isolated preadipocytes were then cultured in a T25 flask at 37 °C and 5% CO_2_, with media refreshment every 1–2 days. Cells were passaged upon reaching 70–80% confluence. To stimulate the browning of rabbit white precursor adipocytes, we supplemented a high glucose medium with dexamethasone (5 mM; Sigma, Shanghai, China), insulin (0.5 mg/mL; Sigma, Shanghai, China), isobutyl-methyl-xanthine (0.5 mM; Sigma, Shanghai, China), rosiglitazone (1 mM; Sigma, Shanghai, China), triiodothyronine (T3) (1 nM; Sigma, Shanghai, China), and isoproterenol (ISO)(10 µM; Sigma, Shanghai, China). The specific culture protocol was as follows: inoculate cells into 12-well plates, 6-well plates, or culture dishes, and incubate at 37 °C in a 5% CO_2_ cell incubator. Replace the growth medium every two days. Once the cell density reaches 80–90%, discard the growth medium, rinse the cells twice with preheated 37 °C PBS, and introduce a new growth medium containing 10 µg/mL insulin, 0.5 mM IBMX, 1 µM dexamethasone, and 50 nM T3 for a two-day culture period. Subsequently, rinse the cells twice with preheated 37 °C PBS and add a growth medium containing 5 µg/mL insulin, 0.5 mM IBMX, 1 µM rosiglitazone, and 50 nM T3 for a four-day culture period. Then, cleanse the cells twice with preheated 37 °C PBS and introduce a growth medium containing 0.5 mM IBMX, 1 µM rosiglitazone, and 50 nM T3 for an additional two-day culture period. Prior to each cell sample collection, ISO should be added to the culture for an advanced incubation period of 4 h. During this time, monitor the morphological changes in white precursor adipocytes and the formation and accumulation of lipid droplets to assess the adipogenic differentiation potential of white precursor adipocytes and lipid droplet formation.

### 4.3. RNA Extraction and RT-qPCR

The Foregene (Fuji Biotechnology Co., Ltd., Chengdu, China) RNA extraction kit was used to extract cell RNA. Then, the reverse transcription kit was used to obtain the cell cDNA. The sample was diluted 2–3 times for qRT PCR detection. All primer information is shown in Table 1.

### 4.4. Oil Red O Staining

After removal of the culture medium, samples were then fixed with 10% formaldehyde at room temperature and rinsed three times with PBS. To stain the lipids in the adipocytes, the cells were treated with filtered Oil Red O solution for 20 min and rinsed twice with PBS. The resulting red-stained lipid droplets were observed microscopically and extracted with isopropanol. The absorbance was measured at 540nm to quantify the residual fat content within the adipocytes.

### 4.5. Cell Transfection

The preadipocytes were inoculated into a 12-well or 6-well plate and transfected with Lipofectamine 3000 (Invitrogen, Carlsbad, CA, USA) according to the manufacturer’s instructions after the cell density reached 90%. The final concentrations of the negative control miRNA mimic (NC miR Mimic), negative control inhibitor (NC inhibitor), and negative control *SON* siRNA (siRNA NC) were 50 nM, 100 nM, and 100 nM, respectively. Each treatment group was repeated three times independently. After transfection, cells were harvested at different time intervals to study the browning of fat.

### 4.6. Western Blotting

CIDEA and UCP1 expression was assessed by Western blot analysis and samples were normalized to β-actin. Protein extraction was blocked with 5% PBS fat-free dried milk at room temperature for 2 h and incubated at 4 °C overnight with anti-CIDEA (1:1000, Abcam), anti-UCP1 (1:1000, Abcam), anti-IgG (1:2000, Abcam), and anti-β-actin (1:3000, Santa Cruz) antibodies, respectively.

### 4.7. Cell Cycle Analysis

After 72 h of transfection, the cells were harvested first and then the cell suspension was digested. Then, the cells were fixed with ethanol (75%) at 4 °C for 4 h; then, the supernatant was discarded and the cells were incubated with an RNA enzyme containing iodide (PI, 40%, Sigma Aldrich). After washing cells with PBS three times, 5 μL of Annexin V-FITC and 10 μL of PI were added and mixed with cells in the dark for 5 min. Finally, the cell cycle was detected by using FACS Calibur (BD Biosciences, USA), and data analysis was conducted through FACS Diva (BD Biosciences, USA). The experiment was repeated three times.

### 4.8. Cell Counting Kit-8 Assay

Cell count kit 8 was used for CCK8 determination (Beyotime, Shanghai, China). In short, cells were seeded on 96-well plates. To test cell viability, a CCK8 reagent was added to each well and incubated at 37 °C for 2 h. Then, the absorption was evaluated with a microplate reader at 450 nm.

### 4.9. Validation of the Target Gene by Double Luciferase Report

miRanda was used to predict the target gene of miR-889-3p. *SON* was found to be the target gene of miR-889-3p. 3′UTR of *SON* containing the miR-889-3p target site was cloned into the Sac I-Xba I site of the pmirGLO vector (Promega, Madison, WI, USA) to construct luciferase report plasmid. Several cloning sites were located downstream of the luciferase gene of firefly. HeLa cells were inoculated into 24-well plates in triplicate. Then, wild-type or mutant pmirGLO *SON* 3 ′ UTR and synthetic miR-889-3p analogs were co-transfected into HeLa cells to achieve a cell density of 70–80%. The activity of firefly luciferase (luc2) was measured 48 h after transfection and, according to TransDetect^®^ Description of the dual luciferase report kit (Transgen, Beijing, China), standardized to Renilla lucifer.

### 4.10. Statistical Analyses

The relative expression amount of all genes and miRNAs in this test was compared by the 2-ΔΔCT method. The experimental results were analyzed by single-factor ANOVA using SAS 9.0 software, and multiple comparisons were performed using the Duncan method. The experimental data were expressed as the mean ± SEM. The difference was significant at * *p* < 0.05.

## 5. Conclusions

In conclusion, our research indicates that miR-889-3p not only stimulates proliferation in rabbit preadipocytes but also promotes the browning of white adipose tissue by targeting *SON*. Further investigations are warranted to elucidate the role and mechanisms of *SON* in the browning process of precursor adipocytes. 

## Figures and Tables

**Table 1:** All primer information.

Gene Name	Primer Sequence (5′-3′)
ACTB	GTGCTTCTAGGCGGACTGTT
	CGGCCACATTGCAGAACTTT
UCP1	CCGGGACAATATGCGAGTGT
	CACAGTCCACAGTCTGCCTT
PRDM16	CCCCGGAAGAACCACGTCTA
	TCTCCACCCTGCTGTAGATGG
C/EBPα	CAAGAACAGCAACGAGTACCG
	GTCACTGGTCAACTCCAGCAC
CIDEA	TAGGGGACAACACGCACTTC
	CTCTGGCGATTCCCGATCTC
PPARα	ACAGGATTGATGCTGCCGAA
	AAACGGATGTGCTGCCCTAA
PGC1α	AAAAGCTTGACTGGCGTCAC
	ACTGCACCACTTGAGTCCAC
TMEM26	TCTATGCCATTCTAATTTTATGGACTTGG
	GATGAAGACGCTGATTCCAATGTTCC
PPARg	GAGGACATCCAGGACAACC
	GTCCGTCTCCGTCTTCTTT
*SON*	ACAGCAGTAGTGCCGAAGTC
	TGGCTCTAGGGCTTTTGCTC
miR-889-3p	TTAATATCGGACAACCATTGT
	GGAACGATACAGAGAAGATTAGC
U6	GGAACGATACAGAGAAGATTAGC
	TGGAACGCTTCACGAATTTGCG

## Data Availability

The data underlying this article will be shared on reasonable request to the corresponding author.

## Supplementary Materials

The following supporting information can be downloaded at https://www.mdpi.com/article/10.3390/ijms242417580/s1.
