# Supplementary figures and images for: miR-889-3p Facilitates the Browning Process of White Adipocyte Precursors by Targeting the SON Gene
Source: Int J Mol Sci. 2023 Dec 17;24(24):17580. doi: 10.3390/ijms242417580 (PMC10743546; doi:10.3390/ijms242417580)

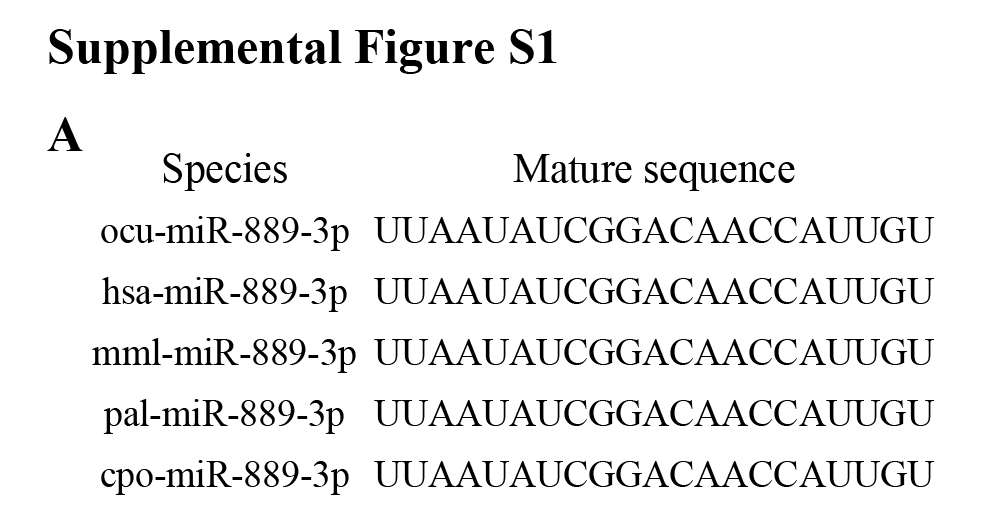

Supplement: Supplementary file 1 [file ijms-24-17580-s001.zip › ijms-2719938-supplementary.jpg]
